# Computational search for the interactors of alternatively spliced TREM2 isoforms

**DOI:** 10.1002/alz.086570

**Published:** 2025-01-03

**Authors:** Junyi Liang, Aditya Aditya Menon, Taylor Tomco, Lynn M. Bekris, Group of GMI staff Dr. Lynn Bekris

**Affiliations:** ^1^ Genomic Medicine Institute, Cleveland, OH USA; ^2^ Case Western University, Cleveland, OH USA; ^3^ Cleveland Clinic Lerner Research Institute, Cleveland, OH USA

## Abstract

**Background:**

Alzheimer’s disease (AD) hallmarks are amyloid plaques and tau tangles. *APOE* and *TREM2* are the strongest genetic risk factors for AD. Triggering receptor expressed on myeloid cells 2 (TREM2) is increasingly recognized to play a central role in amyloid beta clearance and microglia activation in AD. TREM2 has several described ligands, including amyloid beta, apoE and tau. It is known that canonical full‐length TREM2 interacts with DAP12 which in turn activates downstream pathways. One study describes TREM2 interaction with TMEM59 as a regulator TMEM59 protein degradation through cell membrane protein–protein interaction. Three TREM2 alternative splicing isoforms have been described. Little is known about protein–protein interactions with all of three of these TREM2 isoforms. The ***hypothesis*** of our study is that full‐length TREM2 and its alternative splicing isoforms interact with other undescribed proteins. The ***aim*** of this study was to perform a systematic computational search for new interactors of different TREM2 isoforms by employing several state‐of‐the‐art structural bioinformatics tools, from initial large‐scale screening to identify a one‐on‐one corroborative simulation and fine visualization.

**Method:**

ProteinPrompt software random forest method was used to scan mega libraries in the Homo sapien proteome to identify potential interactors of TREM2 isoforms. The top 5 predictions from ProteinPrompt were simulated in PEPPI, in order to verify the authenticity of the interactions. The TREM2 isoforms and their newfound interactors, identified by ProteinPrompt and validated by PEPPI, were then built into heterodimeric complex in AlphaFold Multimer.

**Results:**

CD9, a cell surface glycoprotein involved in cell‐cell adhesion and migration, was identified as a new interactor for two TREM2 isoforms. CALM, a calcium binding protein involved in calcium metabolism, was identified as a new interactor for a third TREM2 isoform.

**Conclusion:**

The predicted interaction of CD9 or CALM with TREM2 isoforms suggests a possible role of TREM2 in cell adhesion and calcium regulation. Both cell adhesion and calcium regulation are two biological systems previously implicated in Alzheimer’s disease.

